# Incidences of Herpes Zoster and Postherpetic Neuralgia in Japanese Adults Aged 50 Years and Older From a Community-based Prospective Cohort Study: The SHEZ Study

**DOI:** 10.2188/jea.JE20140210

**Published:** 2015-11-05

**Authors:** Yukiko Takao, Yoshiyuki Miyazaki, Masayuki Okeda, Fumitake Onishi, Shuichiro Yano, Yasuyuki Gomi, Toyokazu Ishikawa, Yoshinobu Okuno, Yasuko Mori, Hideo Asada, Koichi Yamanishi, Hiroyasu Iso

**Affiliations:** 1 The Research Foundation for Microbial Diseases of Osaka University, Kanonji, Kagawa, Japan; 2 Division of Clinical Virology, Kobe University Graduate School of Medicine, Kobe, Japan; 3 Department of Dermatology, Nara Medical University School of Medicine, Nara, Japan; 4 Public Health, Department of Social Medicine, Osaka University Graduate School of Medicine, Suita, Osaka, Japan

**Keywords:** herpes zoster, postherpetic neuralgia, incidence, prospective cohort study, Japanese adults

## Abstract

**Background:**

Many cross-sectional studies have examined the incidences of herpes zoster (HZ) and postherpetic neuralgia (PHN), but prospective studies in Japanese older adults are lacking. Therefore, we conducted a community-based prospective cohort study to determine the incidence in Japanese adults aged ≥50 years.

**Methods:**

We recruited 12 522 participants from Shozu County, Kagawa Prefecture, between December 2008 and November 2009 and followed participants for 3 years. When a subject presented with symptoms suggestive of HZ, they were examined at collaborating medical institutions and cooperated with onset and recovery surveys (eg, measurement of varicella zoster virus-specific immunity and a pain survey). The hazard ratios (HRs) of HZ and PHN according to sex and age were analyzed by Cox regression analysis with a significance level of 5%.

**Results:**

The incidence of HZ was 10.9/1000 person-years (men: 8.5/1000 person-years; women: 12.8/1000 person-years) and was significantly higher in women than in men (HR 1.5; 95% confidence interval, 1.2–1.8). The incidence of PHN was 2.1/1000 person-years (men: 1.7/1000 person-years; women: 2.4/1000 person-years), with no significant sex differences. A total of 19% of HZ cases progressed to PHN; no sex-specific difference in the proportion of PHN cases was observed.

**Conclusions:**

We clarified the accurate incidences of HZ and PHN in a population of Japanese older adults. These incidences increased with age. HZ incidence was higher in women than in men, while PHN incidence did not differ markedly between the sexes.

## INTRODUCTION

Herpes zoster (HZ) develops when a latent varicella zoster virus (VZV) infection becomes reactivated at a site, such as the dorsal root ganglion, because of aging or decreased immunity and proliferates.^1^ Typical clinical symptoms of HZ are a unilateral rash in the region of distribution of affected nerves accompanied by neuralgia. The rash first appears as pale edematous erythema, which is followed by the formation of vesicles. Pustules, ulcers, and scarring can also develop as the condition worsens. Neuralgia often develops before the rash, and pain can be mild to severe, including pain that disturbs sleep or becomes exacerbated by contact of skin with garments.^2^

When pain persists for 3 months or more after the acute phase of HZ, the condition is called postherpetic neuralgia (PHN), which is a very problematic condition because it considerably decreases quality of life. Currently, there are a growing number of options for palliative treatment, including pharmacotherapy, physical therapy, and nerve blocks, but this condition results in a large economic and psychological burden on the affected person when it persists for an extended period of time.^3^

In Japan, there is no surveillance system to investigate the incidences of HZ and PHN; thus, the incidences of both conditions remain unknown. Currently, the only confirmed incidences are based on cross-sectional analysis of health information from medical institutions.

Because of rapid aging in Japan and elsewhere in the world, epidemiological investigation for the incidences in HZ and PHN are important to estimate the potential health burden in the elderly. Therefore, we decided to conduct a community-based prospective cohort study, which is considered the best design for accurately determining the incidences of HZ and PHN, in adults aged ≥50 years.

## METHODS

The SHEZ (Shozu Herpes Zoster) Study is a community-based prospective cohort study investigating age- and sex-specific incidences of HZ and PHN and risk factors for these outcomes. The methods of this study have been described previously^4^; only a summary is given below.

### Enrollment

Between December 2008 and November 2009, subjects were recruited from among 19 058 Japanese residents aged ≥50 years as of 1 October 2008 in Shozu County (Shodoshima Island and Teshima Island), Kagawa Prefecture.

### Basic information obtained at enrollment

At the time of enrollment, each subject was asked about their birth date, sex, history of HZ (whether they had been diagnosed at a medical institution), underlying diseases, and information about their lifestyle habits (eg, frequency of smoking and drinking).

For subjects who wished to have their immune status measured, an intradermal skin test was conducted to measure VZV-specific cell-mediated immunity using the Varicella Skin Test Kit Antigen “Biken” (The Research Foundation for Microbial Diseases of Osaka University, Kagawa, Japan),^5^ and blood tests were performed to measure VZV-specific cell-mediated immunity (enzyme-linked immunospot assay^6^) and humoral immunity (neutralizing antibody,^7^ glycoprotein-based enzyme-linked immunosorbent assay,^8^ and immune adherence hemagglutination^8^).

### Surveys conducted during the 3 years after enrollment

A telephone survey was conducted with each subject once every 4 weeks to confirm the presence or absence of rash and pain, history of contact with a varicella patient, and history of admission to a clinic or hospital. In addition, subjects were asked to report if they developed a rash or pain before the next call. One objective of this telephone survey was to determine systematically whether a subject had developed HZ during that particular month. The other objective was to raise awareness of HZ among elderly subjects through regular phone calls so that they could be promptly and easily examined at a medical institution for an onset survey if they develop symptoms suggestive of HZ.

### Management of subjects who developed symptoms suggestive of herpes zoster during the 3-year period

Subjects who presented with symptoms suggestive of HZ during the study period were invited to the medical institutions and examined by physicians. When a subject was clinically diagnosed with HZ or possible HZ, their informed consent for the onset survey was obtained, and the following investigation was done by the research team: (1) evaluation of clinical symptoms, (2) blood collection to measure VZV-specific cell-mediated and humoral immunity, (3) crust sampling for virus identification testing (polymerase chain reaction assay),^5^ (4) evaluation of pain, and (5) photographing skin areas with rash and pain.

The surveyed clinical symptoms included information about underlying disease, immunosuppressant use, sites/dates of rash and pain development, unique symptoms of generalized HZ or multiple HZ, and treatments in progress (eg, antiviral agents).

The survey form for evaluation of pain was administered at regular intervals until pain disappeared, for at least 1 week and up to 6 months. It included questions about pain severity, pain distribution, changes in tactile sensation in painful areas, treatment status, and effects on quality of life, such as interference with everyday activities and sleep.

In addition, at 3 months after the onset, subjects provided informed consent for the recovery survey, after which physicians confirmed PHN, motor paralysis, and scarring through a survey of sequelae, and blood was collected to measure VZV-specific cell-mediated and humoral immunity, as in the onset survey.

### Assessment of herpes zoster and postherpetic neuralgia

To calculate the incidences of HZ and PHN, three dermatologists from the Nara Medical University School of Medicine reviewed the information of the onset and recovery surveys and gave a definitive diagnosis of HZ according to the following criteria: (1) when VZV DNA was detected, (2) when the clinical appearance of the rash was typical of HZ (pale edematous erythema or vesicles distributed in aggregates along the nerves), (3) when pain persisted, and (4) when there was a shift in VZV-specific immunity.

Subjects who met the first criteria were generally diagnosed with HZ. If VZV DNA was not detected in a subject that met any of the other criteria, HZ was diagnosed in a comprehensive manner by performing a more rigorous evaluation to determine which of criteria 2–4 were met by the subject.

A diagnosis of PHN was given for subjects who met any of the following criteria: (1) pain that persisted for 3 months or more as indicated in the pain survey, (2) PHN listed in the recovery survey, or (3) subjects with HZ or possible HZ who took the onset survey answered that they were experiencing pain in the area of the rash in a telephone survey 3 months or more after the onset.

### Methods for calculating herpes zoster/postherpetic neuralgia incidence and assessing statistical significance

Incidences of HZ and PHN were expressed per 1000 person-years, and sex-specific and age-specific (age at enrollment) incidences were determined. All subjects who died during the survey were cross-referenced with their certification of residence from the town government and censored at the time of death. Date of loss to follow-up was defined as the date when the subject had not responded to three consecutive monthly telephone surveys, refused the follow-up survey, or could not responded properly to the telephone survey properly due to dementia. The time of HZ onset was defined as the date when rash or pain developed, and the time of PHN development was defined as the date 3 months after HZ onset.

The tests for sex and age differences in the HZ and PHN incidence were performed using Cox regression analysis, and the proportion of PHN among the HZ cases was evaluated using logistic regression analysis. In both cases, the covariates were age and history of HZ when sex-specific differences were assessed, and sex and history of HZ when age differences were assessed.

Analyses were conducted using SAS statistical software package, version 9.2 (SAS Institute Inc., Cary, NC, USA). All statistical tests were two-sided, and *P* values <0.05 were considered statistically significant.

### Standard protocol approvals, registrations, and patient consent

This study was conducted in accordance with the Ethical Guidelines for Epidemiological Research and the Ethical Guidelines for Clinical Studies after obtaining informed consent from subjects. The study was also approved by the ethics committees of the institutions with which the members of this research group are affiliated: The Research Foundation for Microbial Diseases of Osaka University, Osaka University Graduate School of Medicine, National Institute of Biomedical Innovation, and Nara Medical University School of Medicine.

## RESULTS

### Number of subjects enrolled and number who developed herpes zoster and postherpetic neuralgia

A total of 12 522 subjects (5587 men [44.6%] and 6935 women [55.4%]) were enrolled in this study, and the average (standard deviation) age was 68.2 (10.6) years (Table 1). Among these, 1841 subjects (14.7%) indicated they had a history of HZ. The average follow-up period was 2.95 years, and 800 subjects (6.4%) discontinued the study: 637 (5.1%) died, 65 (0.5%) were lost to follow-up, 55 (0.4%) withdrew, 42 (0.3%) moved out, and 1 (0.0%) became demented (follow-up rate: 93.6%). HZ was definitively diagnosed in 401 subjects (139 men [34.7%] and 262 women [65.3%]) at an average age of 69.5 (10.1) years (Table 2). Among the 401 subjects who developed HZ, VZV DNA was detected in 392 (97.8%). VZV DNA was not detected in the remaining 9 subjects, but they were definitively diagnosed with HZ on the basis of the comprehensive assessment of clinical symptoms, severity and duration of pain, and changes in immunity. Among the 401 subjects definitively diagnosed with HZ, 79 (19.7%) developed PHN (28 men [35.4%] and 51 women [64.6%]) at an average age of 72.4 (10.2) years. Among PHN cases, pain persisted for 6 months or more in 35 subjects (44.3%).

**Table 1.** Baseline characteristics of participants

	Men	Women	Total
Number of subjects	5587	6935	12 522
Age distributions, years			
Range	50–100	50–103	50–103
50–59	1505 (26.9)	1685 (24.3)	3190 (25.5)
60–69	1836 (32.9)	1976 (28.5)	3812 (30.4)
70–79	1458 (26.1)	1948 (28.1)	3406 (27.2)
≥80	788 (14.1)	1326 (19.1)	2114 (16.9)
Mean age [SD], years	67.2 [10.1]	68.9 [10.9]	68.2 [10.6]
History of herpes zoster	601 (10.8)	1240 (17.9)	1841 (14.7)
Number of respondents for questionnaires	5507	6845	12 352
Underlying diseases	3369 (61.2)	4205 (61.4)	7574 (61.3)
Hypertension	1734 (31.5)	2175 (31.8)	3909 (31.7)
Hyperlipidemia	290 (5.3)	578 (8.4)	868 (7.0)
Diabetes	656 (11.9)	568 (8.3)	1224 (9.9)
Connective tissue disease	63 (1.1)	197 (2.9)	260 (2.1)
Cancer	222 (4.0)	195 (2.9)	417 (3.4)
Leukemia	3 (0.1)	9 (0.1)	12 (0.1)
Others	82 (1.5)	93 (1.4)	175 (1.4)

SD, standard deviation.

Proportions are shown in parentheses.

**Table 2.** Baseline characteristics in non-herpes zoster cases, herpes zoster cases, and postherpetic neuralgia cases

	Non HZ cases	HZ cases	PHN cases
Number of subjects	12 121	401	79
Sex			
Men	5448 (45.0)	139 (34.7)	28 (35.4)
Women	6673 (55.1)	262 (65.3)	51 (64.6)
Age distributions, years			
Range	50–103	51–98	52–89
50–59	3101 (25.6)	89 (22.2)	14 (17.7)
60–69	3702 (30.5)	110 (27.4)	15 (19.0)
70–79	3277 (27.0)	129 (32.2)	26 (32.9)
≥80	2041 (16.8)	73 (18.2)	24 (30.4)
Mean age [SD], years	68.1 [10.6]	69.5 [10.1]	72.4 [10.2]
History of herpes zoster	1785 (14.7)	56 (14.0)	7 (8.9)
Number of respondents for questionnaires	11 952	400	79
Underlying diseases	7297 (61.1)	277 (69.3)	59 (74.7)
Hypertension	3786 (31.7)	123 (30.8)	27 (34.2)
Hyperlipidemia	839 (7.0)	29 (7.3)	3 (3.8)
Diabetes	1182 (9.9)	42 (10.5)	9 (11.4)
Connective tissue disease	250 (2.1)	10 (2.5)	4 (5.1)
Cancer	396 (3.3)	21 (5.3)	5 (6.3)
Leukemia	11 (0.1)	1 (0.3)	1 (1.3)
Others	171 (1.4)	4 (1.0)	0 (0)

HZ, herpes zoster; PHN, postherpetic neuralgia; SD, standard deviation.

Proportions are shown in parentheses.

### Herpes zoster incidence

The incidence of HZ was 10.9/1000 person-years (Table 3). When sex-specific incidences (men: 8.5/1000 person-years; women: 12.8/1000 person-years) were analyzed using age and history of HZ as covariates, the hazard ratio (HR) was 1.5 (95% confidence interval [CI], 1.2–1.8) for women versus men. When sex differences in the incidence were examined in each age group, the incidence was significantly higher among women than among men aged 50–59 years (HR 1.6; 95% CI, 1.0–2.5, *P* = 0.037) and 60–69 years (HR 1.9; 95% CI, 1.3–2.8). The incidence also tended to be higher among women than among men aged 70 years and older, but this difference was not significant.

**Table 3.** Sex- and age-specific incidence rates of herpes zoster and postherpetic neuralgia per 1000 person-years

Age	Men			Women			Total				Hazard ratio (95% CI)	
			
	Person- years	Cases	Rate per 1000 person- years	Person- years	Cases	Rate per 1000 person- years	Person- years	Cases	Rate per 1000 person- years		vs Men^a^	vs 50–59 years /50–69 years^b^
HZ												
50–59	4547	32	7.0	5110	57	11.2	9656	89	9.2		1.6 (1.0–2.5)^d^	Ref.
60–69	5527	36	6.5	5950	74	12.4	11 477	110	9.6		1.9 (1.3–2.8)^e^	1.1 (0.80–1.4)
70–79	4247	48	11.3	5744	81	14.1	9990	129	12.9		1.3 (0.87–1.8)	1.4 (1.1–1.8)^d^
≥80	2120	23	10.8	3687	50	13.6	5807	73	12.6		1.3 (0.78–2.1)	1.3 (1.0–1.8)^c^
Total	16 441	139	8.5	20 490	262	12.8	36 931	401	10.9		1.5 (1.2–1.8)^f^	
PHN												
50–59	4592	7	1.5	5188	7	1.3	9781	14	1.4	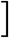	1.0 (0.46–2.0)	Ref.
60–69	5570	7	1.3	6059	8	1.3	11 629	15	1.3			
70–79	4313	6	1.4	5843	20	3.4	10 156	26	2.6	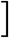	1.6 (0.86–3.0)	2.4 (1.5–3.8)^f^
≥80	2142	8	3.7	3740	16	4.3	5882	24	4.1			
Total	16 617	28	1.7	20 831	51	2.4	37 448	79	2.1		1.3 (0.81–2.1)	

CI, confidence interval; HZ, herpes zoster; PHN, postherpetic neuralgia.

^a^Adjusted for age and history of HZ.

^b^Adjusted for sex and history of HZ.

^c^*P* < 0.10, ^d^*P* < 0.05, ^e^*P* < 0.01, ^f^*P* < 0.001.

When age differences in the incidence were analyzed using sex and history of HZ as covariates, the incidence was highest among subjects aged 70–79 years (12.9/1000 person-years) and those aged ≥80 years (12.6/1000 person-years), while it was lowest among those aged 50–59 years (9.2/1000 person-years). The HR was 1.4 (95% CI, 1.1–1.8) for subjects aged 70–79 years and 1.3 (95% CI, 1.0–1.8, *P* = 0.074) for those aged ≥80 years compared with those aged 50–59 years.

### Postherpetic neuralgia incidence

The incidence of PHN was 2.1/1000 person-years (Table 3). When sex differences in the incidence (men: 1.7/1000 person-years; women: 2.4/1000 person-years) were analyzed using age and history of HZ as covariates, the HR for women versus men was 1.3 (95% CI, 0.81–2.1). When sex differences in incidence were examined in each age group, no significant differences were found.

Because of the small number of PHN cases, we collapsed the four age groups into 50–69 years and ≥70 years when age differences in the incidence were analyzed using sex and history of HZ as covariates. The incidence was highest among subjects aged 80 years and older (4.1/1000 person-years), while it was lowest among those aged 60–69 years (1.3/1000 person-years). The HR was 2.4 (95% CI, 1.5–3.8) for subjects aged ≥70 years compared to those aged 50–59 years.

### Proportion of progression to postherpetic neuralgia

There were 79 PHN cases among 401 HZ cases (Table 4). When sex-specific proportions of PHN (men: 20.1%; women: 19.5%) were analyzed using age and history of HZ as covariates, the OR for women versus men was 0.93 (95% CI, 0.55–1.6).

**Table 4.** The population of postherpetic neuralgia among the herpes zoster cases

Age, years	Men			Women			Total			Prevalence ratio of PHN (95% CI)	
			
	HZ cases	PHN cases	%	HZ cases	PHN cases	%	HZ cases	PHN cases	%	vs Men^a^	vs 50–59 years^b^
50–59	32	7	21.9	57	7	12.3	89	14	15.7	0.51 (0.16–1.7)	Ref.
60–69	36	7	19.4	74	8	10.8	110	15	13.6	0.45 (0.15–1.4)	0.87 (0.40–1.9)
70–79	48	6	12.5	81	20	24.7	129	26	20.2	2.3 (0.85–6.3)^c^	1.4 (0.69–2.9)
≥80	23	8	34.8	50	16	32.0	73	24	32.9	0.88 (0.31–2.5)	2.7 (1.2–5.6)^d^
Total	139	28	20.1	262	51	19.5	401	79	19.7	0.93 (0.55–1.6)	

CI, confidence interval; HZ, herpes zoster; PHN, postherpetic neuralgia.

^a^Adjusted for age and history of HZ.

^b^Adjusted for sex and history of HZ.

^c^*P* < 0.10, ^d^*P* < 0.05.

Further, when age difference in the proportions of PHN was analyzed using sex and history of HZ as covariates, the proportion was highest among subjects aged ≥80 years (32.9%) and lowest among those aged 60–69 years (13.6%), and the OR was 2.7 (95% CI, 1.2–5.6) for subjects aged ≥80 years versus those aged 50–59 years.

## DISCUSSION

In the first attempt of such a study in Japan, we confirmed the incidences of HZ and PHN using a prospective cohort study design. We found that the incidence of HZ was 10.9/1000 person-years, and the incidence of PHN was 2.1/1000 person-years among Japanese residents aged 50 years and older.

Previously reported incidences of HZ and PHN have been determined through cross-sectional analysis methods using the data from health insurance databases,^9^^–^^13^ health information databases,^14^^–^^18^ or independent surveillance databases.^19^^–^^21^ The study periods and subjects (number, age, and race) varied (Table 5), and the reported incidences ranged from 3.2–12.5/1000 person-years for HZ and 0.4–1.5/1000 person-years for PHN (Table 6).

**Table 5.** Summary of previous studies on the incidence of herpes zoster

Country	Study period	Population	Ages of subjects	Data sources	Number of HZ and PHN patients	HZ incidence rate among men/women (/1000PY)	Reference number
The present study Japan (Shozu county)	2009– 2012	12 522 participants	≥50	Data from prospective cohort study	401(HZ) 79(PHN)	8.5/12.8 Higher in women	
Japan (Miyazaki Prefecture)	1997– 2006	The population of the Miyazaki Prefecture (approximately 1.2 million people)	All ages	Medical records	48 388(HZ)	3.67/4.58 Higher in women	15
US (1)	1998– 2004	19 247 subjects of placebo group	≥60	Data from clinical trial	642(HZ) 80(PHN)	10.65/11.79	22
US (2) (Olmsted County, MN)	1996– 2001	Approximately 125 thousand residents	≥22	Medical records	1669(HZ)	3.2/3.9 Higher in women	14
US (3)	2000– 2001	Over 4 million U.S. individuals	All ages	Health insurance	9152(HZ)	2.6/3.8 Higher in women	9
US (4)	2005– 2009	Approximately 51 million insured individuals	≥18	Health insurance	435 378(HZ)	Higher in women	13
Italy	2003– 2005	About 30% of the Italian population	≥15	Medical records	5675(HZ) 350(PHN)	3.82/4.75 Higher in women	16
Holland	2004– 2008	Approximately 167 000 inhabitants	All ages	Medical records	3371(HZ)		17
UK	1994– 2001	Number of patients registered within the British National Health System	All ages	Original surveillance	14 532(HZ)	Higher in women (Except for ages 15–24 years)	19
Germany	2007– 2008	Approximately 69% (in 2007) and 52% (in 2008) of the total German population	≥50	Health insurance	374 645(HZ)	Higher in women	12
Spain (Valencian Community)	2007– 2010	Over 98% of Valencian Community population (approximately 5.1 million inhabitants)	All ages	Medical records	85 586(HZ)	3.86/5.32 Higher in women	18
Australia	2000– 2006	646 000 patients in the Bettering the Avaluation of Care and Health (BEACH) database	≥50	Original surveillance	379(HZ) 57(PHN)		20
Israel	2006– 2010	25% of Israeli population (approximately 2 million individuals)	All ages	Original surveillance	28 977(HZ) 1508(PHN)	Higher in women	21
Taiwan	2000– 2005	98% of Taiwan’s population (22.9 million people in 2006)	All ages	Health insurance	672 782(HZ)	4.72/5.20 Higher in women	10
Korea	2003– 2007	Residence-registration in Korea (49.2 million people in 2007)	All ages	Health insurance	2 431 744(HZ)	Higher in women	11

HZ, herpes zoster; PHN, postherpetic neuralgia; PY, person-years.

**Table 6.** Age-specific incidence rates of herpes zoster and postherpetic neuralgia

Country	Incidence rate (/1000PY)			Age, years																			

				0–4	5–9	10–14	15–19	20–24	25–29	30–34	35–39	40–44	45–49	50–54	55–59	60–64	65–69	70–74	75–79	80–84	85–89	90–94	≥95
The present study	HZ	M+W	10.9											9.2		9.6		12.9		12.6			
Japan (Shozu county)	PHN	M+W	2.1											1.4		1.3		2.6		4.1			
Japan (Miyazaki Prefecture)	HZ	M+W	4.15	2.45		2.86		2.27		1.96		2.53		5.23		6.95		7.84		6.93		5.37	
US (1)	HZ	M+W	11.12													10.79		11.50					
	PHN	M+W	1.38													0.74		2.13					
US (2) (Olmsted County, MN)	HZ	M+W	3.4, 3.6^a^					1.3, 1.6^a^		1.6, 1.9^a^		2.1, 2.3^a^		4.2, 4.7^a^		6.0, 7.1^a^		8.6, 10.0^a^		10.7, 12.0^a^			
US (3)	HZ	M+W	3.2^a^	1.1^a^			1.4^a^			2.0^a^		2.9^a^		4.6^a^		6.9^a^		9.5^a^		10.9^a^			
US (4)	HZ	M+W	4.82				3.37^b^							6.43		7.71	8.43						
Italy	HZ	M+W	—				1.71	1.82	1.91	2.25	1.95	2.51	3.06	4.15	5.63	6.90	7.11	8.22	8.56	7.97	6.13		
Holland	HZ	M+W	4.75	3.28												9.31	11.32						
UK	HZ	M	—	1.62			2.13		1.99				3.99				7.75		9.58				
		W	—	2.31			2.11		2.45				5.91				9.84		11.04				
Germany	HZ	M+W	9.60^a^											6.21^a^	7.59^a^	8.94^a^	10.70^a^	11.34^a^	12.15^a^	12.53^a^	12.58^a^	13.19^a^	
	PHN	M+W	0.43–1.33											0.05– 0.50	0.22– 0.76	0.26– 0.98	0.35– 1.39	0.37– 1.70	0.78– 2.19	0.96– 2.63	0.97– 2.39	1.02–2.51	
Spain (Valencian Community)	HZ	M+W	4.29	2.01			2.27			2.33		2.86		5.84		8.57		9.76		9.38			
Australia	HZ	M+W	9.67^a^	1.4^a^										6.52^a^		8.58^a^		14.50^a^		15.61^a^			
	PHN	M+W	1.45^a^											0.73^a^		1.21^a^		2.35^a^		3.16^a^			
Israel	HZ	M+W	3.46	1.91			2.00		2.35		2.62		4.44		7.48		10.23		11.45		8.97		
Taiwan	HZ	M+W	4.97	1.64–3.51								5.18		8.36		11.09		12.32		10.21			
Korea	HZ	M+W	12.54	2^c^	3^c^	4^c^	4^c^	5^c^	7^c^	7^c^	8^c^	9^c^	14^c^	20^c^	25^c^	31^c^	35^c^	40^c^	42^c^	38^c^	31^c^	23^c^	20^c^

HZ, herpes zoster; M, men; PHN, postherpetic neuralgia; PY, person-years; W, women.

^a^Extrapolated for the national census.

^b^Age range was between 18 and 49 years.

^c^Incidence rate estimated from graph.

All of these previous studies, except for an American randomized clinical trial,^22^ were cross-sectional studies, and they used different methods for the ascertainment and diagnosis of HZ and PHN. We adopted methods similar to those used in the American clinical trial, and thus we may be able to compare our results with the findings of that trial. The subjects of the American clinical trial were retired military personnel recruited at 22 medical institutions in the United States between 1998 and 2004. In that study, the incidence of HZ was 11.1/1000 person-years and the incidence of PHN was 1.4/1000 person-years. Compared with these figures, we found a similar incidence of HZ and a slightly higher incidence of PHN.

It is likely that the decreased VZV-specific cell-mediated immunity of elderly adults is the reason why HZ and PHN incidences were high among patients in this age group. A previous paper published by our research team showed that the average major axes of the erythema and edema induced by a intradermal skin test, both indices of VZV-specific cell mediated immunity, was smaller when the subjects were older, and that the incidences of HZ and PHN were higher when the average major axes of the erythema and edema were smaller.^5^ Based on these findings, we were able to validate the hypothesis that the susceptibility to HZ and PHN increases with age because of weakened VZV-specific cell-mediated immunity. Many other studies have reported that VZV-specific cell-mediated immunity decreases with age.^23^^–^^27^

Cell-mediated immunity seems be more deeply involved in preventing HZ onset than humoral immunity because VZV is highly cytophilic and spreads cell to cell after invading the body. In this study, we actually found that VZV-specific humoral immunity increases with age, which was inversely related to the changes in cell-mediated immunity.^28^ Previous studies, however, found no increase in humoral immunity with age.^24^^–^^26^

There was no sex difference with regard to PHN incidence, but HZ incidence was significantly higher in women than in men. Previous studies have yielded similar results, but the reason for this finding remains unclear.^10^^,^^13^^–^^16^^,^^18^^,^^19^^,^^22^ We previously showed that lowered cell-mediated immunity contributes to the higher incidence of HZ among elderly adults.^6^ However, the reason for the higher incidence of HZ among women than among men is also unclear because there is no sex difference in cell-mediated immunity.^23^^–^^27^

To identify risk factors for developing HZ, we collected information on a range of potential risk factors, including lifestyle habits, underlying diseases, and VZV-specific cell-mediated immunity and humoral immunity. Previous studies found that risk factors for HZ included family history, depression, negative life events, socioeconomic status, organ transplantation, HIV treatment, antineoplastic medications, and histories of diabetes, leukemia, and cancer, while inhibiting factors for HZ included smoking, excellent self-rated health, having intimate friends, and black race.^9^^,^^18^^,^^21^^,^^29^^–^^31^ Therefore, some factors involved in the higher incidence in women than in men may be lifestyle habits or psychosocial factors unique to women. Among the sex-specific incidences in this study, the most marked sex difference in incidence was observed among subjects aged 60–69 years (HR 1.9; 95% CI, 1.3–2.8); thus, we may focus on lifestyle characteristics of women in this age group in future studies regarding risk factors for HZ.

Strengths of the present study are (1) use of a community-based approach rather than a medical institution-based approach; (2) enrollment of a wide range of subjects aged ≥50 years living in Shozu County, Kagawa Prefecture, without excluding certain professions or underlying diseases; and (3) confirmation of HZ onset through not only clinical diagnosis but also use of samples of crusts, vesicles, and blood to assess changes in VZV DNA detection, VZV-specific humoral immunity, and VZV-specific cell-mediated immunity for each case to determine the onset accurately.

We conducted the study on an island of Shozu County, which is located within a distance of only 30 or 60 minutes by high-speed craft and ferry from the main island of Japan. Therefore, island residents regularly commute between the main and smaller islands. According to the Infectious Diseases Weekly Report Kagawa, there are outbreaks of chickenpox (ie, varicella) among children in Shozu County every year, which affects HZ incidence among elderly adults,^32^^,^^33^ as well as periodic pandemics like in other regions across the nation. Therefore, our estimated incidences of HZ and PHN can be generalized to other populations.

According to the summary data from the 2010 Population Census of Japan released by the Statistics Bureau of the Ministry of Internal Affairs and Communications, there are approximately 55.6 million individuals aged ≥50 years in Japan. If the age- and sex-specific HZ and PHN incidences obtained through this study were applied to the entire population of Japan to estimate the number of new cases each year, there would be approximately 590 000 cases of HZ and approximately 110 000 cases of PHN. As the population continues to grow older, the incidence of HZ may increase further. Preventing HZ is important because PHN substantially decreases quality of life in elderly adults.
